# Subgingival Microbiota Profile in Association with Cigarette Smoking in Young Adults: A Cross-Sectional Study

**DOI:** 10.3390/dj9120150

**Published:** 2021-12-11

**Authors:** Krešimir Bašić, Kristina Peroš, Zrinka Bošnjak, Ivana Šutej

**Affiliations:** 1 Department of Pharmacology, School of Dental Medicine, University of Zagreb, 10000 Zagreb, Croatia; basic@sfzg.hr (K.B.); isutej@sfzg.hr (I.Š.); 2 Department for Clinical and Molecular Microbiology, University Hospital Centre Zagreb, 10000 Zagreb, Croatia; zbosnjak@kbc-zagreb.hr

**Keywords:** smoking, subgingival bacteria, microbiota, young adults

## Abstract

While smoking is recognized as one of the factors for the development and progression of periodontal diseases, a relation between the composition of the subgingival microbiota and smoking is yet to be elucidated. The aim of this study was to investigate the prevalence of subgingival bacteria in young smokers and non-smokers without clinical signs of periodontal disease. In this cross-sectional study, performed at the Department of Pharmacology, School of Dental Medicine, University of Zagreb, we enrolled 32 periodontally healthy smokers and 32 non-smokers, aged 25–35 years old. The number of oral bacteria and the prevalence of particular bacteria were assessed for each subject. Subgingival plaque samples were collected with sterile paper points from two first molars for microbiological analyses with MALDI-TOF mass spectrometry. In smokers, a significantly higher prevalence of *Actinomyces odontolyticus* was observed compared to non-smokers, and a significantly lower prevalence of *Streptococcus sanguinis* was observed compared to non-smokers. Smoking affects the composition of subgingival microbiota, either via depletion of beneficial bacteria or the increase in pathogenic bacteria.

## 1. Introduction

Smoking is recognized as one of the major factors for the development and early onset of periodontal disease [1,2]. A large number of studies have shown clinical implications of smoking on periodontium, but little is known about the mechanisms, especially on oral bacteria, which are the main etiological agent in the development of periodontitis. There are several possible mechanisms of how smoking can alter living conditions for bacteria in the mouth: an increase in the acidity of saliva [3], reducing the availability of oxygen [4], the effect on bacterial adherence to the mucous membranes [5], toxins from cigarette smoke can have antibiotic activity [6], or an alteration of the immune response [7]. Smoking favors the spectrum of anaerobic bacteria [8,9,10], which is the pool of periodontal pathogens, though there are studies that have shown the opposite [11]. There is a significant influence of smoking on the microbiological flora of the oral cavity in developed periodontal disease [1,9,10,12,13,14], whether it is an increased number of pathogenic bacteria, a greater prevalence of individual pathogens, or that microbial flora of smokers and non-smokers is simply different by species or groups of bacteria. Even though there are major studies that have investigated the impact of smoking on the subgingival microbial composition in patients with developed periodontal disease, there are only few such studies on periodontally healthy subjects, and the results of these studies are very different and often contradictory [8,12,15,16,17,18]. Some studies have confirmed the association of smoking and microbial flora in periodontally healthy subjects, regardless of whether it is a higher number of different colonies of periodontal pathogens [19] or a reduced number of protective bacteria [9,20]. Although there are studies that have not confirmed the association of smoking and subgingival microbial flora [12,16,17], the results of most studies confirm that smoking affects the subgingival microbial flora in an as yet unknown way [18]. The reasons for these contrasting results can be many, such as: the variety of methods of identification of bacteria, different target populations, different sampling techniques, or insufficient exclusion of various confounding factors. To overcome some of the reasons detected, this study was designed with a uniform sample, considering age, periodontal health, and smoking status. The aim of this study was to investigate whether smoking affects the prevalence of subgingival bacteria in young adults without clinical signs of periodontal disease.

## 2. Materials and Methods

### 2.1. Study Design

This cross-sectional study was performed at the Department of Pharmacology, School of Dental Medicine, University of Zagreb, Zagreb, Croatia. The study protocol was approved by the Ethics Committee of the Faculty of Dental Medicine, University of Zagreb. All respondents confirmed their informed consent to participate in the study. Their identities were kept confidential by assigning them with numeric codes at enrolment and keeping the informed consents and questionnaires separately. The study was performed in accordance with the World Health Organization Declaration of Helsinki [21].

### 2.2. Subjects

**Figure 1:**
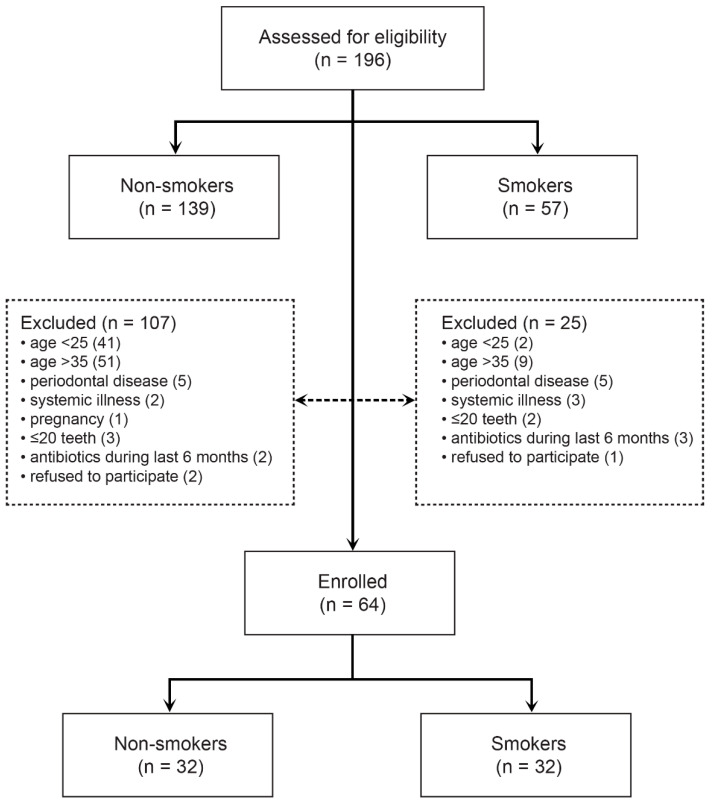
Study flow.

The target population were young adults aged 25 to 35 years of both genders who smoked at least 1 pack/day with good oral and systemic health and without clinical signs of periodontal disease. The control group consisted of non-smokers from the same age group. The non-inclusion criteria for both groups were the presence of periodontitis, the existence of systemic diseases and medication, pregnancy, less than 20 teeth in the mouth, taking antibiotics during the six months before the enrolment, and periodontal or orthodontic therapy during the same period. Fifty-seven (57) smokers and one hundred and thirty-nine (139) non-smokers were assessed for eligibility, and a consecutive sample of patients was selected in the order of arrival at the clinic for the exam (Figure 1). All subjects were instructed not to brush their teeth, eat, or drink anything but water 1 h before clinical examination and sampling for bacterial analyses.

### 2.3. Sample Size

The power analysis was accomplished by Hintze J. (2013) PASS 12. NCSS. The determination of sample size was performed on a bacteria-based analysis. A minimum of 32 patients per group is required if a difference of 25% in prevalence of bacteria between the two groups is to be detected at a significance level of a *p* = 0.05 with a power of 80%, using a conservative two-tailed testing approach. This difference between the two groups was considered clinically significant according to previous studies [22]. Hence, the inclusion of at least 64 patients in the study would yield the adequate statistical power for group comparisons.

### 2.4. Microbiological Sampling and Analyses

Samples for microbiological analysis were obtained with sterile endodontic paper points (Paper Points #40, DiaDent Europe B.V., Almere, The Netherlands).

Paper points were set into the sulcus of mesial surfaces of the two first molars (16 and 46), removed after 30 s, immersed in a transport medium (thioglycolate broth for anaerobic bacteria and saline for aerobic bacteria), and transported to a laboratory for microbiological analysis.

The broth solutions were cultured on Columbia agar and blood agar at 38 °C in an anaerobic atmosphere for 48 h for anaerobic bacteria and at 37 °C in an aerobic atmosphere for 48 h for aerobic bacteria. Growth of colonies was processed according to the general algorithm for Gram-positive or Gram-negative bacteria [23]. All isolates were subjected to matrix-assisted laser desorption/ionization time-of-flight mass spectrometry (MALDI-TOF-MS) analysis (Bruker Daltonik GmbH, Bremen, Germany). For MALDI-TOF-MS analysis, a protein extraction from bacterial isolates was performed as previously described [24,25].

The extract was analyzed with MALDI Biotyper 3.1 (Bruker Daltonik GmbH, Bremen, Germany), and the spectra obtained were compared to the MALDI Biotyper database that contains 5989 protein spectra. The Bruker Bacterial Test Standard was used for calibration. Protein profiles with a mass-to-charge ratio (*m*/*z*) of 2000–20,000 Da were produced based on a laser frequency of 60 Hz, 240 laser shots of each spectrum, and 40 shot steps.

### 2.5. Clinical Examination

Periodontal indexes used in the definition of targeted populations were determined using a standard periodontal probe (PCP-15, Hu-Friedy, Chicago, IL, USA) and dental mirror. Periodontal examination was performed by the same experienced dental practitioner who measured the approximate plaque index (API), bleeding on probing (BoP), probing depth (PD), gingival recession (RE), and clinical attachment level (CAL). Oral hygiene was assessed by the plaque index by O’Leary et al. (Plaque Control Record) [26]. The existence of plaque was examined on four surfaces of each tooth (vestibular, oral, mesal, distal), based on which the incidence of plaque was expressed according to the formula: API = plaque number/number of all places tested. BoP was examined on four surfaces of each tooth and expressed according to the formula: BoP = number of bleeding sites/number of all tested sites. When the probing was applied, the force of 0.25 N was applied, and the depth of the probe was defined as the distance from the free margin of the gingiva to the stop of the probe at the bottom of the pocket. Gingival recession was defined as the distance from the enamel–cement joint to the free margin of the gingiva and determined on the vestibular and oral side of each tooth. The level of clinical attachment was defined as the distance from the enamel–cement joint to the bottom of the pocket and was calculated by adding the value of the probing depth and the gingival recession of the individual tooth surfaces. For the purposes of research, the average values of all the mentioned periodontal parameters with regard to the number of teeth examined or dental surfaces were determined. Periodontally healthy subjects were considered to have at least 20 teeth with PD less than 4.0, BoP less than 0.25, and none of which had a pocket depth greater than or equal to 4 mm.

### 2.6. Statistical Data Analysis

The main hypothesis was tested using robust regression analysis. We tested the normality of residuals using Shapiro–Wilk and D’Agostino Omnibus tests and checked for multicollinearity by inspecting the variance inflation factors (<2.5) and condition numbers (>15). The analysis of the differences in the prevalence of particular bacteria was performed using the Mann–Whitney U test, and relative risks (RR) with their 95% confidence intervals for having particular bacteria were calculated. The level of statistical significance was set at *p* < 0.05 and confidence intervals at 95%.

Confidence scores greater than 2.0 were considered secure species identification, scores of 1.7–2.0 were considered intermediate identification, and scores of less than 1.7 were considered unreliable identification.

Statistical data analysis was performed using the R Core Team (2018), R: A language and environment for statistical computing, R Foundation for Statistical Computing, Vienna, Austria.

## 3. Results

In this study, 32 participants were enrolled in each group. The two groups were well-balanced in terms of age, frequency of dental exams, and periodontal indices (Table 1).

Non-smokers harbored a median (IQR) of 5 (2–7) different species, and smokers harbored 6 (1–8) different species. This difference was not significant (Mann–Whitney test, U = 455, Z = −0.78, *p* = 0.437). At the genus level, significant differences between smokers and non-smokers were not found (Table 2).

In smokers, a significantly higher prevalence of *Actinomyces odontolyticus*: 8 (25%; 95% CI 11–43%), was observed compared to non-smokers: 1 (3%; 95% CI 1–16%) (RR = 8.0, 95% CI 1.13–170.05, Fisher exact test, *p* = 0.026) (Table 3), and a significantly lower prevalence of *Streptococcus sanguinis*: 6 (19%; 95% CI 7–37%), compared to non-smokers: 13 (41%; 95% CI 24–60%) (RR = 0.46, 95% CI 0.17–1.13, Fisher exact test, *p* = 0.049) (Table 4).

## 4. Discussion

In this study, special attention was paid to the uniformity of the sample. All subjects were matched by age, periodontal status, and smoking status, which were inclusion criteria for further analysis.

A statistically significant prevalence of *A. odontolyticus* bacteria was found in smokers. This species is best known for its role in the development of caries of the root and the development of pulpitis [27]. The main feature of this species is the ability to adhere to the tooth surface and to coagulate with other bacteria [28,29]. *A. odontolyticus*, coupled with *Veillonella parvula*, makes the purple complex according to Socransky et al. [30] and belongs to the early colonists of the tooth surface and subgingival space. As the authors explain, the purple complex serves as a bridge to the orange and in some places red complexes, and thus this species is also associated with periodontal disease. In studies comparing the microbiological profile of healthy subjects with profiles of those with some form of periodontal disease, there is a greater prevalence of *A. odontolyticus* bacteria in periodontal patients [31,32,33], although there are studies that have not found such a connection [34]. Further research is needed to clarify the influence of smoking on *A. odontolyticus* and its influence on the onset of periodontal disease.

One of the findings in this research was a statistically significant higher prevalence of *S. sanguinis* in non-smokers compared to smokers. Shchipkova et al. [35] in their study of 15 smokers and 15 non-smokers with chronic periodontitis obtained a statistically significant lower prevalence of *S. sanguinis* in smokers. Mason et al.’s [8] study comparing 200 periodontally healthy smokers and non-smokers also showed a lower prevalence of *S. sanguinis* in smokers. Tanner et al. [36] compared healthy subjects with subjects who developed gingivitis and with subjects with initial periodontitis. The prevalence of *S. sanguinis* was the highest in healthy subjects, while in subjects with initial periodontitis, no *S. sanguinis* was recorded at all. Mager et al. [37] indicate a higher prevalence of *Streptococcus* in non-smokers compared to smokers and periodontal patients. Similar results were obtained from Thomas et al. [38] and Lie et al. [39]. Wu et al. [11] and Kumar et al. [15] found the opposite results at the genus level—the *Streptococcus* genus was more common in smokers. In these studies, there are no data separate available for *Streptococcus* species, so there is no indication of the role of *S. sanguinis* in this distribution.

*S. sanguinis* is a species that according to Socransky et al. [30] falls into the yellow spectrum, and with *Streptococcus mitis* and *Streptococcus oralis*, belongs to the earliest colonizers of the tooth surface and subgingival space. According to many authors, *S. sanguinis* belongs to benign, protective bacteria [40,41,42,43]. Stingu et al. [40], in a study of 33 subjects with aggressive periodontitis and 20 healthy subjects in the control group, established a correlation between subjects with aggressive periodontitis with lower prevalence of *S. sanguinis*. Teughels et al. [43], in an in vitro study, showed the influence of *S. sanguinis* on reduced adhering ability of *Aggregatibacter actinomycetemcommitans*. *S. sanguinis* produces hydrogen peroxide which inhibits adherence and growth of periodontal pathogens *A. actinomycetemcommitans* [42,43] and *Porphyromonas gingivalis* [44]. A similar effect of *S. sanguinis* bacteria was studied in relation to the known cariogenic pathogen *S. mitis* [41,45]. *A. actinomycetemcommitans*, on the other hand, has the ability to produce a bateriocin that can kill *S. sanguinis* [46], which confirms the exceptional complexity of an ecosystem such as a biofilm on the surface of the tooth, where the winner ultimately determines whether a disease will develop. The results of our research show that smokers, although healthy and without periodontal pathogens, might have a greater risk of developing periodontal disease because of the depletion of beneficial bacteria.

Regarding prevalence of bacteria at the genus level in the non-smokers and smokers, this particular sample showed a higher prevalence of the *Fusobacteria* genus in smokers (38% in smokers, 18% in non-smokers), although the difference was not statistically significant. Species of the *Fusobacteria* genus, often associated with periodontal disease [30], in many studies of smokers with advanced periodontal disease, show increased prevalence [35,47,48]. Species from this genus are not the most important periodontal pathogens but play a very important role in the formation of subgingival biofilm. This biofilm acts as a bridge between early colonizers and periodontal pathogens, and also fulfils a local immunosuppressive role [49]. The lack of statistical significance in our results could be explained by the limitations regarding our culturing method and microbiological analysis.

There are a few major limitations in this study that could be addressed in future research. First is its cross-sectional design. We were unable to establish the temporal order between smoking and the incidence of particular bacteria. Consequently, we could not test any causal hypothesis. Second, as all of our data were collected in a single institution in the highly urbanized and more prosperous capital of Croatia, our study results should not be generalized to apply to the entire Croatian population, or to specific populations living in rural and poorer regions with lower access to primary dental care, different prevalence of smoking, and different dietary habits. Although we performed the power analysis before the data collection, our sample size was relatively small, and the precision and reliability of our findings are limited as well. One of the limitations is the analysis method we used for determining different bacteria species. Even though MALDI-TOF is recognized as a valid scientific method, there are other culture-independent methods that can detect larger amounts of different bacteria species.

In conclusion, smoking could be responsible for the depletion of beneficial bacteria and the increase in potentially pathogenic bacteria, but further studies with a larger sample are needed to enlighten our understanding of the effect of smoking on the subgingival bacteria.

## Figures and Tables

**Table 1:** Participants’ sociodemographic and clinical characteristics.

	Non-Smokers (*n* = 32)		Smokers (*n* = 32)	
Gender				
women	15	(46.9)	13	(40.6)
men	17	(53.1)	19	(59.4)
Age (years), median (IQR)	31	(26–34)	30	(27–32)
Dental exams at least once a year	20	(62.5)	21	(65.6)
Periodontal indices				
approximal plaque index	0.18	(0.13–0.22)	0.15	(0.10–0.22)
bleeding on probing	0.25	(0.16–0.30)	0.23	(0.08–0.31)
periodontal pocket depths (mm)	2.42	(2.32–2.49)	2.50	(2.26–2.65)
gingival retraction (mm)	0.18	(0.11–0.26)	0.14	(0.03–0.34)
clinical attachment level	2.47	(2.35–2.55)	2.56	(2.30–2.72)
Duration of smoking (years), median (IQR)			12	(8–15)

Data are presented as number (percentage) of participants if not stated otherwise. Abbreviations: IQR = interquartile range.

**Table 2:** Prevalence of bacteria at the genus level.

	Non-Smokers (*n* = 32)		Smokers (*n* = 32)		*p*	φ
*Actinobacteria*	15	(46.9)	20	(62.5)	0.315	0.16
*Proteobacteria*	10	(31.3)	9	(28.1)	>0.999	0.03
*Bacteroidetes*	5	(15.6)	6	(18.8)	>0.999	0.04
*Firmicutes*	28	(87.5)	25	(78.1)	0.509	0.12
*Fusobacteria*	6	(18.8)	12	(37.5)	0.164	0.21

Data are presented as number (percentage) of participants. *p* = statistical significance of the prevalence in non-smokers and smokers calculated using the Fisher exact test only for bacteria with the frequency of ≥3; φ = phi coefficient of association.

**Table 3:** Prevalence of anaerobic bacteria.

	Non-Smokers (*n* = 32)		Smokers (*n* = 32)		*p*	φ
*Veillonella parvula*	8	(25.0)	9	(28.1)	>0.999	0.08
*Fusobacterium nucleatum*	5	(15.6)	8	(25.0)	0.536	0.12
***Actinomyces odontolyticus***	**1**	**(3.1)**	**8**	**(25.0)**	**0.026**	**0.32**
*Actinomyces oris*	12	(37.5)	7	(21.9)	0.274	0.17
*Parvimonas micra*	4	(12.5)	5	(15.6)	>0.999	0.00
*Fusobacterium canifelinum*	1	(3.1)	4	(12.5)	0.355	0.18
*Capnocytophaga granulosa*	2	(6.3)	3	(9.4)	>0.999	0.06
*Veillonella atypica*	2	(6.3)	3	(9.4)	>0.999	0.06
*Capnocytophaga gingivalis*	1	(3.1)	3	(9.4)	0.613	0.13
*Gemella morbillorum*	5	(15.6)	2	(6.3)	0.672	0.11
*Propionibacterium acnes*	2	(6.3)	2	(6.3)		
*Gemella haemolysans*	1	(3.1)	2	(6.3)		
*Veillonella dispar*	1	(3.1)	2	(6.3)		
*Prevotella intermedia*	3	(9.4)	1	(3.1)	0.613	0.13
*Capnocytophaga ochracea*	2	(6.3)	1	(3.1)		
*Actinomyces meyeri*	1	(3.1)	1	(3.1)		
*Fusobacterium periodonticum*	1	(3.1)	1	(3.1)		
*Prevotella nigrescens*	1	(3.1)	1	(3.1)		
*Prevotella spp*	1	(3.1)	1	(3.1)		
*Atopobium parvulum*			1	(3.1)		
*Campylobacter showae*			1	(3.1)		
*Capnocytophaga sputigena*			1	(3.1)		
*Eubacterium brachy*			1	(3.1)		
*Fusobacterium naviforme*			1	(3.1)		
*Gemella bergeri*			1	(3.1)		
*Lactobacillus salivarius*			1	(3.1)		
*Leptotrichia wadei*			1	(3.1)		
*Prevotella buccae*			1	(3.1)		
*Prevotella loescheii*			1	(3.1)		
*Prevotella dentalis*	2	(6.3)				
*Prevotella melaninogenica*	2	(6.3)				
*Aggregatibacter aphrophilus*	1	(3.1)				
*Campylobacter concisus*	1	(3.1)				
*Leptotrichia trevisanii*	1	(3.1)				
*Prevotella denticola*	1	(3.1)				
*Veillonella rogosae*	1	(3.1)				
No bacteria	9	(28.1)	10	(31.3)		

Data are presented as number (percentage) of participants if not stated otherwise. Bacteria are sorted by the prevalence in smokers. Significant difference between groups in bold font. Abbreviations: *p* = statistical significance of the prevalence in non-smokers and smokers calculated using the Fisher exact test only for bacteria with the frequency of ≥3; φ = phi coefficient of association.

**Table 4:** Prevalence of aerobic bacteria.

	Non-Smokers (*n* = 32)		Smokers (*n* = 32)		*p*	φ
*Streptococcus oralis*	18	(56.3)	13	(40.6)	0.317	0.16
*Streptococcus mitis*	9	(28.1)	14	(43.8)	>0.999	0.07
*Streptococcus salivarius*	3	(9.4)	8	(25.0)	0.184	0.21
***Streptococcus sanguinis***	**13**	**(40.6)**	**6**	**(18.8)**	**0.049**	**0.24**
*Rothia mucilaginosa*	3	(9.4)	6	(18.8)	0.474	0.14
*Streptococcus intermedius*	3	(9.4)	6	(18.8)	0.474	0.14
*Streptococcus parasanguinis*	5	(15.6)	5	(15.6)	>0.999	0.00
*Streptococcus gordonii*	2	(6.3)	5	(15.6)	0.426	0.15
*Rothia dentocariosa*	3	(9.4)	4	(12.5)	>0.999	0.05
*Staphylococcus epidermidis*	3	(9.4)	4	(12.5)	>0.999	0.16
*Neisseria flavens*	2	(6.3)	3	(9.4)	>0.999	0.06
*Staphylococcus hominis*			3	(9.4)	0.238	0.22
*Streptococcus pneumoniae*	6	(18.8)	2	(6.3)	0.257	0.19
*Haemophilus parainfluenze*	5	(15.6)	2	(6.3)	0.426	0.15
*Neisseria mucosa*	1	(3.1)	2	(6.3)		
*Neisseria bacilliformis*			2	(6.3)		
*Streptococcus anginosus*			2	(6.3)		
*Rothia aeria*	1	(3.1)	1	(3.1)		
*Streptococcus cristatus*	1	(3.1)	1	(3.1)		
*Aggregatibacter aphrophilus*			1	(3.1)		
*Streptococcus vestibularis*			1	(3.1)		
*Staphylococcus capitis*	2	(6.3)				
*Campylobacter showae*	1	(3.1)				
*Enterococcus faecalis*	1	(3.1)				
*Neisseria elongata*	1	(3.1)				
*Neisseria macacae*	1	(3.1)				
*Staphylococcus aureus*	1	(3.1)				
*Staphylococcus lugdunensis*	1	(3.1)				
*Streptococcus cristatus*	1	(3.1)				
*Streptococcus mutans*	1	(3.1)				
No bacteria	4	(12.5)	6	(18.8)		

Data are presented as number (percentage) of participants if not stated otherwise. Bacteria are sorted by the prevalence in smokers. Significant difference between groups in bold font. Abbreviations: *p* = statistical significance of the prevalence in non-smokers and smokers calculated using the Fisher exact test only for bacteria with the frequency of ≥3; φ = phi coefficient of association.

## Data Availability

The data presented in this study are available on request from the corresponding author. The data are not publicly available due to the privacy of participants.
